# Toward Understanding the Functional Role of *Ss-riok-1*, a RIO Protein Kinase-Encoding Gene of *Strongyloides stercorali*s

**DOI:** 10.1371/journal.pntd.0003062

**Published:** 2014-08-07

**Authors:** Wang Yuan, James B. Lok, Jonathan D. Stoltzfus, Robin B. Gasser, Fang Fang, Wei-Qiang Lei, Rui Fang, Yan-Qin Zhou, Jun-Long Zhao, Min Hu

**Affiliations:** 1 State Key Laboratory of Agricultural Microbiology, Key Laboratory of Development of Veterinary Diagnostic Products, Ministry of Agriculture, College of Veterinary Medicine, Huazhong Agricultural University, Wuhan, China; 2 Department of Pathobiology, School of Veterinary Medicine, University of Pennsylvania, Philadelphia, Pennsylvania, United States of America; 3 Faculty of Veterinary Science, The University of Melbourne, Parkville, Victoria, Australia; University of South Florida, United States of America

## Abstract

**Background:**

Some studies of *Saccharomyces cerevisiae* and mammals have shown that RIO protein kinases (RIOKs) are involved in ribosome biogenesis, cell cycle progression and development. However, there is a paucity of information on their functions in parasitic nematodes. We aimed to investigate the function of RIOK-1 encoding gene from *Strongyloides stercoralis*, a nematode parasitizing humans and dogs.

**Methodology/Principal Findings:**

The RIOK-1 protein-encoding gene *Ss-riok-1* was characterized from *S. stercoralis*. The full-length cDNA, gDNA and putative promoter region of *Ss-riok-1* were isolated and sequenced. The cDNA comprises 1,828 bp, including a 377 bp 5′-UTR, a 17 bp 3′-UTR and a 1,434 bp ORF encoding a protein of 477 amino acids containing a RIOK-1 signature motif. The genomic sequence of the *Ss-riok-1* coding region is 1,636 bp in length and has three exons and two introns. The putative promoter region comprises 4,280 bp and contains conserved promoter elements, including four CAAT boxes, 12 GATA boxes, eight E-boxes (CANNTG) and 38 TATA boxes. The *Ss-riok-1* gene is transcribed throughout all developmental stages with the highest transcript abundance in the infective third-stage larva (iL3). Recombinant *Ss*-RIOK-1 is an active kinase, capable of both phosphorylation and auto-phosphorylation. Patterns of transcriptional reporter expression in transgenic *S. stercoralis* larvae indicated that *Ss*-RIOK-1 is expressed in neurons of the head, body and tail as well as in pharynx and hypodermis.

**Conclusions/Significance:**

The characterization of the molecular and the temporal and spatial expression patterns of the encoding gene provide first clues as to functions of RIOKs in the biological processes of parasitic nematodes.

## Author Summary

Parasitic nematodes cause serious global health problems and enormous economic losses. Control of these parasites is difficult due to their complicated life cycle and the lack of knowledge of their developmental biology at the molecular level. Protein kinases are key molecules regulating a range of biological processes of organisms. The atypical protein kinase RIOK-1 was reported to be indispensable in yeast, as well as in free-living nematode *Caenorhabditis elegans*, but little is known about its function in parasitic nematodes. In the present study, we investigate the RIOK-1 encoding gene (*Ss-riok-1*) and its predicted protein *Ss*-RIOK-1 from parasitic nematode *Strongyloides stercoralis* which causes canine and human diseases. We found that *Ss*-RIOK-1 has high sequence identities (50–65%) to its homologues from both vertebrates and invertebrates. It also has abilities of phosphorylation and auto-phosphorylation *in vitro*. *Ss-riok-1* transcript is present in all stages of *S. stercoralis* with more abundance in the parasitic stages than in the free-living stages, along with the gene expression in neuron system of post free-living L1 and body muscle of iL3, indicating that it plays important role in the development and infection of *S. stercoralis*. The findings have important implications for understanding the function of RIOK-1 in the development of parasitic nematodes.

## Introduction

*Strongyloides stercoralis* is a parasitic nematode infecting human beings and dogs, and causes a fatal, disseminated hyperinfection in immuno-compromised patients [1], [2]. The life cycle of *S. stercoralis*, like other members of *Strongyloides* and related genera, is more complicated than that of most obligatory parasitic nematodes. *S. stercoralis* can execute both parasitic and free-living generations of development. Parasitic female adults (P Female) live in the host intestine and produce sexually differentiated eggs by mitotic parthenogenesis. Eggs of *S. stercoralis* hatch in the host intestine and in immune-competent hosts, newly hatched post parasitic first-stage larvae (PP L1) are passed in the feces. Once in the environment, female post-parasitic L1 can either develop directly (homogonically) to infective third stage larvae (iL3) and infect a host or develop heterogonically to free-living female adults (FL Female). Male PP L1s invariably develop via the heterogonic route to the free-living male adults (FL Male). Post-free-living L1 (PFL L1) produced by FL Female and FL Male are all female and develop to iL3. Female PP L1 of *S. stercoralis* may develop precociously to autoinfective L3 (aiL3) within the intestine, penetrate the intestinal wall, invade the somatic tissues and ultimately establish as a new generation of P Female in the primary host intestine. This process of autoinfection may proceed for sequential generations in an immuno-compromised host, with geometric expansion of parasite numbers and involvement of multiple body tissues, possibly leading to a fatal outcome for such immuno-compromised hosts [3].

In contrast to the relative wealth of information on the complex life cycle of this parasite, the understanding of molecular factors regulating its developmental biology is limited. Elucidating the functions of the essential genes that regulate the development and reproduction of *S. stercoralis* could facilitate the discovery of novel interventions for strongyloidiasis and other related parasitic nematode diseases.

Protein kinases are a large group of enzymes that are crucial in the regulation of a wide range of cellular processes, including cell-cycle progression, transcription, DNA replication and metabolic functions [4]. Based on their structures, protein kinases can be classified into eukaryotic protein kinases (ePKs) and atypical protein kinases (aPKs) [5]. The ePKs contain a conserved catalytic domain that phosphorylates enzymes of signal transduction pathways that regulating many biological processes. The aPKs are active kinases containing kinase domains with limited sequence similarity to the conserved catalytic domain of ePKs. According to their characteristics in their kinase domains and functions in different biological processes, the aPKs have been divided into 13 families, one of which contains the RIO kinases. There are currently four members of the RIOK family, RIOK-1, RIOK-2, RIOK-3 and RIOK-B [6], [7]. RIOK-1 and RIOK-2 are strongly conserved from archaea to human, whereas RIOK-3 is only found in metazoans, and RIOK-B is restricted to eubacteria [6]. RIOK-1 controls cell cycle progression and chromosome maintenance in yeast [8]–[10] and participates in aspects of ribosomal biogenesis including 20S rRNA cleavage and maturation of ribosomal small subunits in both yeast and human cells [8], [11]. The genome of the free-living nematode *Caenorhabditis elegans* also encodes RIOK-1 [12]. A large-scale double-stranded RNA interference (RNAi) study of *C. elegans* showed that the silencing of *Ce-riok-1* leads to embryonic lethality and arrest of larval development [13]–[17]. This finding suggests that RIOK-1 is essential for development and growth of nematodes. In spite of the functional importance of this molecule in *C. elegans*, there is no published information on the functions of RIOK-1 in any related parasitic nematodes, other than DNA sequence characterization and bioinformatic analyses of RIOK encoding genes of the ovine parasitic nematodes *Trichostrongylus vitrinus* [18] and *Haemonchus contortus* [19] These studies revealed that *riok-1* of *T. vitrinus* is transcribed at the highest level in iL3 and proposed that *riok-1* of *H. contortus* is a potential drug target. However, almost nothing is known about the function of this gene for any parasite.

Transgenesis, which is very useful for functional genomic studies in *C. elegans* [20], was successfully established in *S. stercoralis* [21], [22], thus providing us with a technical platform to investigate the functions of genes in this parasite [21]–[23]. Because of the potential of this parasitic nematode for functional genomic studies, we aimed to isolate and characterize *Ss-riok-1* and to explore the temporal and spatial expression patterns of this gene, with a view towards uncovering its function. Information on the function of *Ss-riok-1* will contribute to an evaluation of RIOKs as potential targets of drugs directed against *S. stercoralis* and related parasitic nematodes.

## Materials and Methods

### Ethics statement

The *S. stercoralis* (UPD strain) was maintained in prednisolone-treated Beagles in accordance with protocol (Permit Number: SYXK-0029) approved by the Committee on the Ethics of Animal Experiments of Hubei Province. The care and maintenance of animals were in strict accordance with the recommendations in the Guide for the Regulation for the Administration of Affairs Concerning Experimental Animals of P.R. China.

### Parasite maintenance and culture

The UPD strain of *S. stercoralis* was maintained in prednisolone-treated dogs and cultured as described [24], [25]. RNA and genomic DNA were extracted from iL3s concentrated from charcoal coprocultures using the Baermann funnel technique [26] after 7–10 days of incubation at 22°C. The iL3s were washed several times with a sterile buffered saline called BU buffer [24], [27] to reduce bacterial contamination. Free-living adult *S. stercoralis* for micro-injection were isolated from charcoal coprocultures using the Baermann funnel, incubated for two days at 22°C and then placed on Nematode Growth Medium (NGM) agar plates seeded with *Escherichia coli* OP50 [24].

### DNA and cDNA preparation

Total genomic DNA was extracted from ∼10,000 iL3 larvae using a small-scale sodium proteinase K extraction [28] followed by mini-column (Promega) purification. Total RNA of *S. stercoralis* was extracted from ∼30,000 iL3 by TRIzol reagent extraction (Life Technologies). RNA yields were estimated spectrophotometrically (NanoDrop Technologies, Thermo). Total 5′-ends cDNA and 3′-ends cDNA were synthesized by Smart RACE Kit (BD Bioscience) following the manufacturer's protocol; cDNAs were stored at −20°C.

### Isolation of *Ss-riok-1* cDNA and promoter region

Degenerate primers 1F and 2R (Table S1) were designed based on the alignment of *riok-1* homologues of *H. contortus* (GenBank accession no. HQ198854.1), *C. elegans* (NM_001026399) and an EST (BI324299.1) from *Strongyloides ratti*. A 210 bp fragment was amplified from the cDNA synthesized from total RNA extracted from *S. stercoralis* iL3s. This PCR product was cloned into the pMD-19T vector (Takara Biotechnology) and sequenced. Based on the isolated sequence, two gene-specific primers (designated 3F and 4R) were designed (Table S1). Using pairs of gene-specific primers and adaptor primers, two partially overlapping cDNA fragments were produced separately from total RNA from *S. stercoralis* iL3s by 5′- and 3′- RACE. After sequencing the two partial cDNAs, five gene-specific primers were designed to amplify the 5′- and 3′- terminal regions of the *Ss-riok-1* cDNA (Table S1). The complete cDNA of *Ss-riok-1* was assembled according to the sequence obtained through 5′- and 3′-RACE PCR. Then a pair of primers with restriction sites (Ss-riok1-*Bam*HI and Ss-riok1-*Xho*I, Table S1) were designed to amplify the coding region of *Ss-riok-1* using the following cycling conditions: initial 94°C, 5 min; then 94°C, 30 s, 60°C, 30 s, 72°C, 2 min for 30 cycles; final extension at 72°C for 10 min. The PCR product was then cloned into pMD19-T vector (Takara Biotechnology) and sequenced.

To isolate the promoter sequence, four genomic DNA libraries were constructed employing Genome-Walker Kit (BD Bioscience), following the manufacturer's instructions. Briefly, genomic DNA of *S. stercoralis* was digested with four restriction enzymes *Dra*I, *Eco*RV, *Pvu*II, *Stu*I, respectively. Then, each of the four digested products was purified by phenol/chloroform extraction [29] and linked to an adapter provided in the kit, producing four libraries. Touch-down PCR was performed using one adapter primer with one gene-specific primer and the following protocol: 7 cycles for 94°C, 25 s, 72°C, 3 min; 32 cycles for 94°C 25 s, 67°C 3 min; and final extension at 67°C, 7 min. The PCR products from the four libraries were examined separately on agorose gels, and the products were gel-purified, cloned into pMD19-T vector (Takara Biotechnology) and sequenced. To isolate the entire promoter sequence, two primers (Ss-rio1-PstI and Ss-rio1-AgeI, Table S1) located in the 5′- and 3′-ends, respectively, were designed and used to amplify the merged sequence using the following protocol: initial 94°C, 5 min; then 94°C, 30 s, 60°C, 30 s, 72°C, 4 min 30 s for 30 cycles; final extension at 72°C for 10 min. The resultant PCR product containing the promoter of *Ss-riok-1* in its entirety was cloned into pGMD19-T vector (Takara Biotechnology) and sequenced and then sub-cloned into pAJ01 [29].

### Bioinformatic and phylogenetic analyses

The sequence of *Ss-riok-1* was compared by BLASTx [30] with sequences in non-redundant databases from NCBI (http://www.ncbi.nlm.nih.gov/) to confirm the identity of genes isolated. The translation of cDNA of *Ss-riok-1* into predicted amino acid sequences was performed by free software Bioedit (http://www.mbio.ncsu.edu/BioEdit/bioedit.html#downloads). The protein motifs of *Ss*-RIOK-1 were identified by scanning the databases PROSITE [31] (www.expasy.ch/tools/scnpsit1.html) and Pfam [32] (www.sanger.ac.uk/Software/Pfam/). *Ss*-RIOK-1 was aligned with the homologues from selected species using the program MAFFT 7.0 [33] (http://mafft.cbrc.jp/alignment/software/), and the functional domains and subdomains were identified in the protein alignment. Promoter elements in the 5′-UTR were predicted using the transcription element search system Matrixcatch (http://www.gene-regulation.com/cgi-bin/mcatch/MatrixCatch.pl) [34].

For phylogenetic analysis, the amino acid sequences of 27 homologues were retrieved from GenBank databases and the alignment of protein sequences was carried out by Clustal X [35] and manually adjusted. The species selected were nine nematodes, including *Ascaris suum* (ERG87084.1), *Brugia malayi* (EDP30009.1), *Caenorhabditis briggsae* (CAP24959.2), *Caenorhabditis elegans* (CCD67367.1), *Caenorhabditis remanei* (XP_003098834.1), *Haemonchus contortus* (ADW23592.1), *Loa loa* (XP_003135673.1), *Trichostrogylus vitrinus* (CAR64255.1), *Wuchereria bancrofti* (EJW88234.1), and 14 non-nematode species, including *Aedes aegypti* (XP_001661999.1), *Arabidopsis thaliana* (NP_851100.1, AAM65700.1, NP_180071.1), *Canis familiaris* (XP_535878.1), *Danio rerio* (NP_998160.1), *Drosophila melanogaster* (NP_648489.1), *Homo sapiens* (EAW55210.1, NP_113668.2), *Mus musculus* (NP_077204.2), *Oryza sativa* (BAC79649.1), *Pan troglodytes* (XP_527225.2), *Pongo abelii* (CAH93232.1), *Rattus norvegicus* (NP_001093981.1, AAH79173.1), *Saccharomyces cerevisiae* (CAA99317.1), *Xenopus laevis* (NP_001116165.1), *Xenopus tropicalis* (XP_004915351.1). The phylogenetic analysis was conducted using the neighbor-joining (NJ), maximum parsimony (MP) and maximum likelihood (ML) methods based on Jones-Taylor-Thornton (JTT) model in the MEGA v.5.0 [36]. Confidence limits were assessed by bootstrapping using 1,000 pseudo-replicates for NJ, MP and ML, and other settings were obtained using the default values in MEGA v.5.0 [36]. A 50% cut-off value was implemented for the consensus tree.

### Transcriptional analysis of *Ss-riok-1*

The *S. stercoralis* PV001 line, derived from a single female worm of the UPD strain [37], was maintained and cultured as described previously [24], [25], [38]. *S. stercoralis* PV001 developmental stages were isolated using established methods [37], [39] and included: free-living females (FL Female), post free-living first-stage larvae (PFL L1), infective third-stage larvae (iL3) (heterogonically developed), *in vivo* activated third-stage larvae (L3+), parasitic females (P Female), post-parasitic first-stage larvae (PP L1), and post-parasitic third-stage larvae (PP L3). Transcript abundances were quantified using RNAseq [39]. Briefly, raw reads were aligned to *S. stercoralis* genomic contigs (6 December 2011 draft; ftp://ftp.sanger.ac.uk/pub/pathogens/HGI/) using the program TopHat2 v.2.0.9 (http://tophat.cbcb.umd.edu/) [40], employing the Bowtie2 aligner v.2.1.0 (http://bowtie-bio.sourceforge.net/bowtie2/index.shtml) [41] and SAMtools v.0.1.19 (http://samtools.sourceforge.net/). Transcript abundances were calculated using Cufflinks v.2.0.2 (http://cufflinks.cbcb.umd.edu/) as fragments per kilobase of coding exon per million fragments mapped (FPKM), with paired-end reads counted as single sampling events [42]. FPKM values for coding sequences (CDS), ±95% confidence intervals, were calculated for each gene using Cuffdiff v.2.0.2. Significant differences in FPKM values between developmental stages and p-values were determined using Cuffdiff v.2.0.2, a program with the Cufflinks package [43], [44]; *p*-values <0.05 were considered statistically significant.

### Protein expression and purification

A full-length cDNA of *Ss-riok-1* was amplified by PCR using primers Ss-riok1-*Bam*HI and Ss-riok1-*Xho*I (Table S1). The PCR product was then cloned into pMD19-T and sequenced, and further subcloned into the vector pGEX-4T-1. The insert of the recombinant plasmid pGEX-4T-1-*riok-1* was sequenced and its open-reading frame (ORF) encoding the fusion protein GST-*Ss*-RIOK-1 was confirmed [45]. This recombinant vector was then used to transform *E. coli* (*Transetta*; Transgene) cells for protein expression. The bacterial cells were diluted 1∶100 into new LB/Amp^+^ medium, after 3 h of growth at 37°C. The bacteria were induced with IPTG (1∶1000), grown at 28°C and 150 rpm/min overnight and then concentrated by centrifugation at 10000 rpm/min for 2 min. The bacteria were re-suspended in 50 mM Tris-Cl with 0.1 M NaCl, passed through a 0.45 µm filter and loaded onto a 1 mL *GST rap 4B* affinity columns (GE Healthcare). The bound *Ss*-RIOK-1 was eluted with 50 mM Tris-HCl, 40 mM reduced glutathione, pH 8.0. The elution was concentrated using a Ultra-15 50 KD centrifugal filter devices (Millipore). The final concentration was 1 mg/mL. As a control, *E. coli Transetta* cells were transformed with null pGEX-4T-1 vector, incorporating a GST tag. The GST protein was purified using the same method as described above.

### Kinase assays

All assays were performed in 20 µL reaction volumes containing 25 mM Tris pH 7.5, 50 mM NaCl and 2 mM MgCl_2_ [46]. 10 µg purified GST-*Ss*-RIOK-1 were added into the autophosphorylation reaction; 2 µg GST-*Ss*-RIOK-1 and 9 µg myelin basic protein (MBP) were added to each phosphorylation reaction. In the control group, the GST-*Ss*-RIOK-1 was replaced with GST. All components were mixed prior to the addition of 1 µCi [γ^32^P] ATP.

### Transformation constructs and transformation of *S. stercoralis*

To make the plasmid for transgenesis, the promoter region of *Ss-riok-1* was digested with restriction enzymes *Pst*I and *Age*I (Thermo) and gel-purified by Tiangen Gel purification kit (Tiangen Biotech). The purified product was then subcloned into the promoter-less vector pAJ01 [29] to create a plasmid pRP1 (Fig. S1). The constructs was extracted by TIANpure Midi Plasmid Kit (Tiangen Biotech) and then was diluted to 30 ng/µL and stored at −20°C.

Adult FL Female *S. stercoralis* were transformed by gonadal micro-injection using an established approach [21]. Briefly, 30 ng/µL of plasmid *Ss-riok-1p::gfp::Ss-era-1t* (pRP1) were injected into the distal gonads of individual worms. Single females transformed with pRP1 were then paired with one or two FL adult males on an NGM+OP50 plate and incubated at 22°C for egg laying. F1 progeny were screened for fluorescence at 24, 48 and 72 h, respectively, after microinjection. *S. stercoralis* larvae were screened for expression of GFP fluorescent reporter transgenes using an Olympus SZX12 stereomicroscope with epifluorescence. Worms with GFP expression were examined in detail using an Olympus BX60 compound microscope equipped with Nomarski Differential Interference Contrast (DIC) optics and epifluorescence (Olympus America Inc.). Specimens were immobilized on a 2% agarose pad (Lonza), anesthetized using 20–50 mM levamisole (Sigma-Aldrich), and imaged using a digital camera (Spot RT Color, Model 2.2.1) and associated image analysis software (Diagnostic Instruments, Inc.) [37]. All images were processed using Photoshop CS 5.0. Image-processing algorithms, primarily brightness and contrast adjustments, were all applied in linear fashion across the entire image.

## Results

### Characterization of the *Ss-riok-1* cDNA

**Figure 1:**
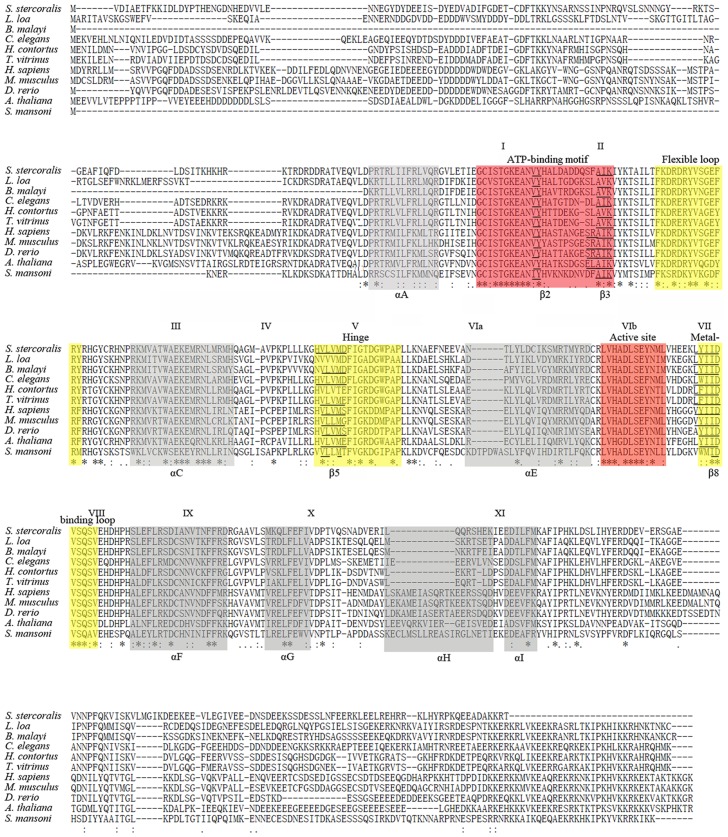
Alignment of the inferred amino acid sequences of *Strongyloides stercoralis Ss*-RIOK-1 with RIOK-1s from nine other species. The nine selected species are *Loa loa* (XP_003135673.1, *Ll*-RIOK-1), *Brugia malayi* (EDP30009.1, *Bm*-RIOK-1), *Caenorhabditis elegans* (CCD67367.1, *Ce*-RIOK-1), *Haemonchus contortus* (ADW23592.1, *Hc*-RIOK-1), *Trichostrogylus vitrinus* (CAR64255.1, *Tv*-RIOK-1), *Homo sapiens* (NP_113668.2, *Hs*-RIOK-1), *Mus musculus* (NP_077204.2, *Mm*-RIOK-1), *Danio rerio* (NP_998160.1, *Dr*-RIOK-1), *Arabidopsis thaliana* (NP_180071.1, *At*-RIOK-1), *Schistosoma mansoni* (XP_002573653.1, *Sm*-RIOK-1). Alpha-helices A-I or beta-sheet structures are highlighted with light grey and marked under the alignment. The subdomains I-XI are marked above the alignment. Functional domains including ATP-binding motif (red), flexible loop (yellow), hinge (yellow), active site (red) and metal binding loop (yellow) are highlighted and labeled above the alignment. Asterisks indicate identical residues.

The full-length cDNA of *Ss-riok-1* (GeneBank Accession No. KJ701282) is 1828 bp in length, including a 5′-UTR of 377 bp, a 17 bp 3′-UTR followed with the polyadenylation signal and a coding sequence of 1,434 bp encoding 477 amino acids. Neither a first nor a second spliced leader sequence (SL1 and SL2, respectively) was identified. In the protein sequence predicted from the gene, the RIOK-1 motif “LVHADLSEYNTL” [9] was identified (Fig. 1). The *Ss*-RIOK-1 shares high sequence identity (50–65%) to RIOK-1s from a diverse range of organisms, including vertebrates, amphibians, fish, plants and nematodes, with the highest identity (65%) to *As*-RIOK-1 from *A. suum*.

Alignment of the amino acid sequences of *Ss*-RIOK-1 with the homologues from selected species (Fig. 1) shows that the conserved regions include the ATP binding motif (sub-domains I and II), the flexible loop, the hinge region (subdomain V), the active site (sub-domain VIb), the metal binding loop (DFG loop, subdomains VII and VIII) and other features of RIOK-1s, such as the C termini of ATP-biding motif G-x-[ILV]-S-T-G-K-E and the altered I-D-V-[SAQ] in the metal-biding motif of *Ss*-RIOK-1. The key residues “Asp” and “Asn” essential for protein kinase activity in the active sites of RIOK-1s, which are involved in catalytic function and are conserved in all ePKs [4], [9]. The amino acid sequences in regions external to these functional subdomains were more divergent than the sequences within them (Fig. 1).

### Relationship of *Ss*-RIOK-1 with orthologues from other species

**Figure 2:**
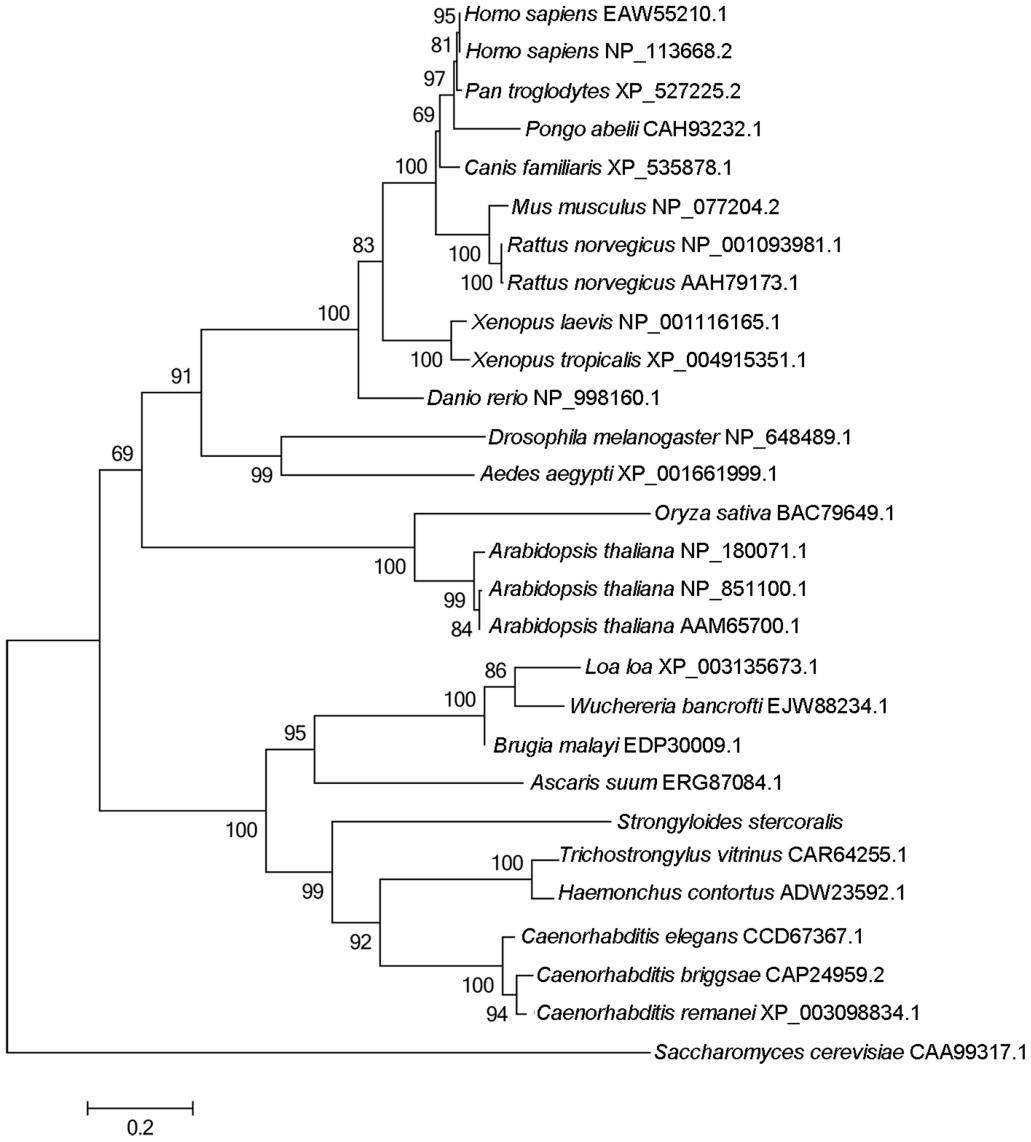
The Neighbor-joining tree of *Strongyloides stercoralis Ss*-RIOK-1 with 27 homologues from 23 selected species. These species contain nine nematode species, two plant species, two insects, three fish and amphibian species and six mammalian species. The RIOK-1 from *Saccharomyces cerevisiae* (CAA99317.1) is used as the outgroup. GenBank accession numbers of the homologous sequences are listed beside the species name. Bootstrap values are displayed above or below the branches.

Results of phylogenetic analyses (Fig. 2) showed that there is concordance in topology among the MP, ML and NJ trees. *Ss*-RIOK-1 groups with orthologues from clade V nematodes [47] with strong (99%) nodal support. The RIOK-1s from parasitic nematodes representing clade III [47] grouped together with strong (95%) support; all nematode RIOK-1s formed a cluster with absolute support to the exclusion of 17 RIOK-1s from 13 non-nematode species. Among the 17 RIOK-1s representing 13 non-nematode species, RIOK-1s from plants, mammals or insects each grouped together, respectively with high bootstrap support (99–100%). The RIOK-1s from other vertebrates, including fish and amphibians, grouped with the RIOK-1s from mammals with strong bootstrap support respectively (100%).

### Genetic structure of *Ss-riok-1* and comparison with orthologues from *C. elegans* and *H. contortus*

**Figure 3:**
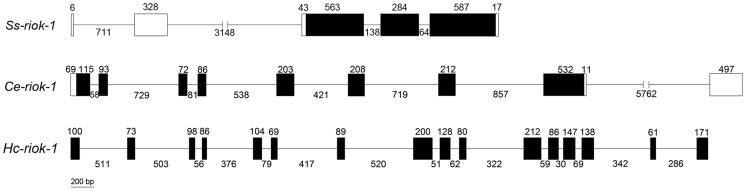
The gene structure of *Ss-riok-1* with comparison to its homologues from *Caenorhabditis elegans* and *Haemonchus contortus*. Black boxes indicate the exons, with the numbers above indicating the length of exon. Introns are indicated by slanted lines between the exons, with the numbers indicating the intron length. The 5′- and 3′- untranslated regions (UTR) of *Ss-riok-1* and *Ce-riok-1* are indicated with white boxes, with the numbers above the box indicating the length of the UTR.

The genomic DNA representing *Ss-riok-1* (GeneBank Accession No. KJ701282) is 5889 bp in length. The 377 bp 5′-UTR from cDNA is interrupted by two large introns of 711 bp and 3148 bp in length, respectively. The coding sequence of *Ss-riok-1* encompasses 1,636 bp, containing three exons of 284–587 bp in size and two introns of 64 bp and 138 bp in size, respectively. The 17 bp 3′-UTR of *Ss-riok-1* follows the third exon of the coding sequence (Fig. 3). Comparison with *Ce-riok-1* (MO1B12.5a, sequences were retrieved from WormBase) from *C. elegans* and *Hc-riok-1* from *H. contortus* [19] showed that these two homologues contain more introns than *Ss-riok-1*. The coding sequence of *Ce-riok-1* contains eight exons of 72–532 bp in size and seven introns of 58–857 bp in size, whereas *Hc-riok-1* has 16 exons of 61–200 bp in size and 15 introns of 30–520 bp in size [19].

### Analysis of the predicted *Ss-riok-1* promoter

The isolated 5′-upstream region of the start codon of *Ss-riok-1* is 4,280 bp in size (GeneBank Accession No. KJ701282). Bioinformatic analysis of transcriptomic and genomic data from *S. stercoralis* revealed that the region between *Ss-riok-1* and the upstream gene was 6,854 bp. The gene upstream of *Ss-riok-1* encoded a putative falvin domain-containing protein and is transcribed in the opposite orientation of *Ss-riok-1*. The putative promoter region of this gene was 3,665 bp; the 4,280 bp DNA region upstream of the start codon of *Ss-riok-1* overlapped by 1,091 bp with the flavin domain-containing protein-encoding gene.

Comparison between the isolated 5′-upstream region of *Ss-riok-1* and the homologous region in *Ce-riok-1* (4,242 bp upstream of the start code of *Ce-riok-1* retrieved from WormBase) showed a sequence identity of 42.7% (Fig. S2). The putative promoter regions of both genes are A+T rich, with the A+T content of 81.1% for *Ss-riok-1* and 68.1% for *Ce-riok-1*, respectively. Further analysis failed to detect CpG islands in either promoter region, but found a GC box (GGCGG) in the promoter region of *Ce-riok-1* that is absent from that of *Ss-riok-1*. This analysis highlighted several promoter elements, including 38 TATA boxes, four CAAT (CCAAT) or inverse CAAT (ATTGG), 12 GATA (WGATAR), 19 inverse GATA (TTATC) and eight E-boxes (CANNTG) in the promoter region of *Ss-riok-1*. With the exception of the GC-boxes, CAAT boxes and inverse CAAT boxes, there are generally fewer such elements in the promoter region of *Ce-riok-1* than in that of *Ss-riok-1*. There are 10 CAAT (CCAAT) or inverse CAAT (ATTGG), one GC-box, two GATA (WGATAR), seven inverse GATA (TTATC), seven E-boxes (CANNTG) and six TATA boxes in the promoter region of *Ce-riok-1*. The four nucleotides preceding the start codon (ATG) are AAGG for *Ss-riok-1* and AAAC for *Ce-riok-1*. The AAAC sequence observed in *Ce-riok-1* differs from the adenine tract AAAA more frequently seen in *C. elegans* genes [48]. The predicted promoter elements are scattered across the promoter regions of the two genes, with no apparent pattern to their distribution.

### Transcriptional analysis of *Ss-riok-1*

**Figure 4:**
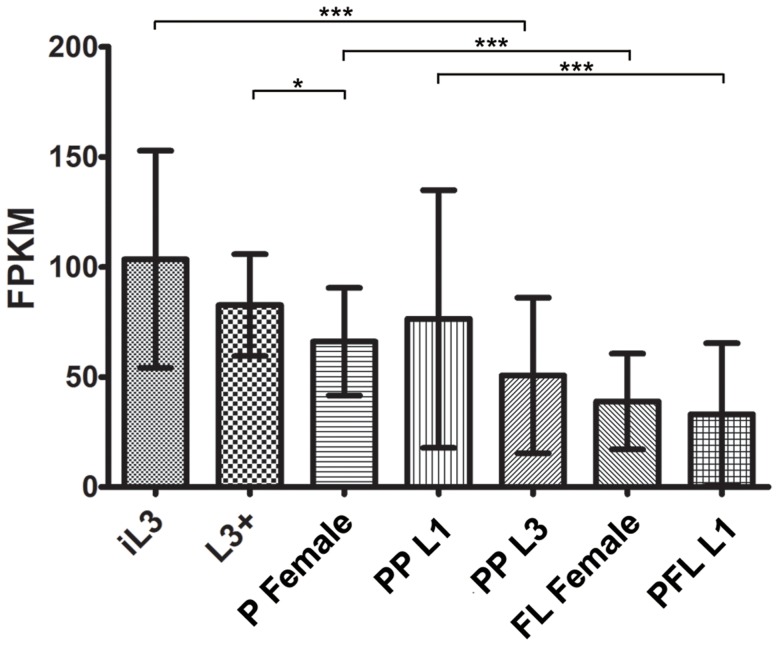
Transcriptional profiles of *Ss-riok-1*. Transcript abundances were determined for the C-terminal coding region of *Ss-riok-1*, in seven developmental stages: parasitic females (P Female), post-parasitic first-stage larvae (PP L1), post-parasitic third-stage larvae (PP L3), free-living females (FL Female), post free-living first-stage larvae (PFL L1), infectious third-stage larvae (iL3), and in vivo activated third-stage larvae (L3+). Transcript abundances were calculated as fragments per kilobase of coding exon per million mapped reads (FPKM). Bracket with 1 star represent the significant difference (*p<0.05*), brackets with 3 stars represent the significant difference (*p*<0.01) in transcript abundances between the two selected stages. Error bars represent 95% confidential intervals.

*Ss-riok-1*-specific transcripts were detected in all developmental stages of *S. stercoralis* examined (Fig. 4). Abundance of these transcripts increases significantly during the transition from PFL L1 to iL3, and remains at a high level in the host-derived L3+. L3+ develop to the parthenogenetic P female during their migration in the host and reach the intestine; a significant decrease with the transcripts abundance of *Ss-riok-1* (*p*<0.05) was found during migration and development. The reduced abundance of *Ss-riok-1* transcripts during development of PP L1s to FL females was also detected. The abundance of *Ss-riok-1* transcripts in iL3 is significantly greater than in PP L3 (*p*<0.001). By contrast, the abundance of *Ss-riok-1* transcripts are significantly higher in P female and PP L1 than in FL female and PFL L1, respectively (*p*<0.001).

### Protein kinase activity of recombinant *Ss*-RIOK-1

**Figure 5:**
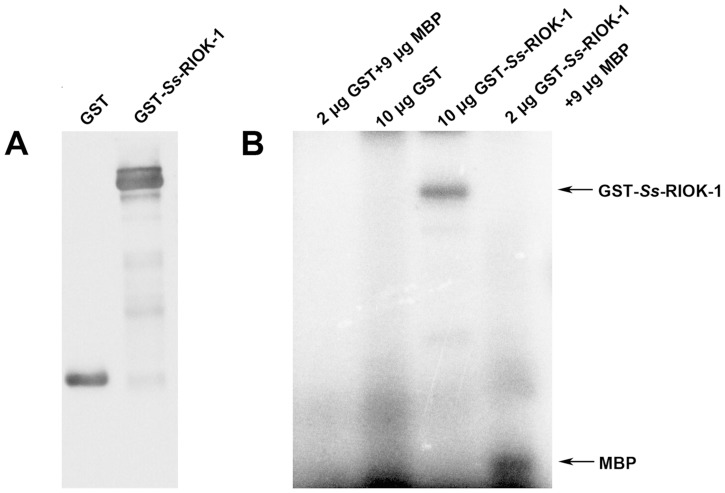
Autophosphorylation and phosphorylation activity of recombinant GST-*Ss*-RIOK-1. A, Western-blot of GST-*Ss*-RIOK-1 (89 kDa) and GST (28 kDa) are immunoprecipitated with GST antibody. B, Kinase assay showing autophosphorylation and phosphorylation activities of recombinant GST-*Ss*-RIOK-1 (89 kDa). Substrate in phosphorylation assay is MBP (18 kDa). Amounts of each protein in the reaction are indicated in the labels. Purified GST constitutes the negative control.

The activities of many protein kinases include phosphorylation and auto-phosphorylation. It is reported that RIOK-1 could also phosphorylate the common protein kinase substrate MBP as well as RIOK-1 itself [49]. To assess the kinase activity of *Ss*-RIOK-1, recombinant GST-*Ss*-RIOK-1 with a GST tag (designated GST-*Ss*-RIOK-1) was expressed in *E. coli* (Fig. 5A). Purified recombinant GST-*Ss*-RIOK-1 incubated with [γ^32^P] ATP only or in the presence of MBP showed radioactive signals associated with the *Ss*-RIOK-1 and MBP, respectively, indicating that the GST-*Ss*-RIOK-1 is capable of both phosphorylation and auto-phosphorylation (Fig. 5B).

### Localisation of *Ss*-RIOK-1 expression

**Figure 6:**
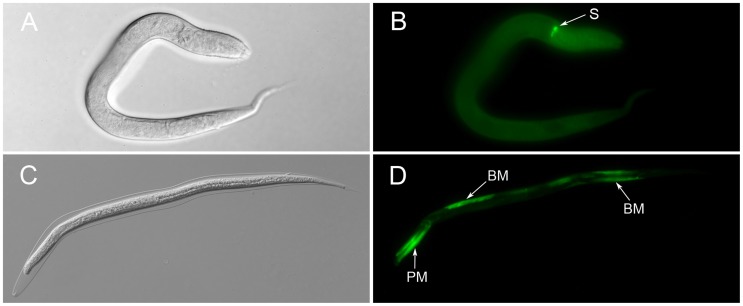
The anatomical expression pattern of *Ss-riok-1* in the early post-free-living first stage larvae. (A, B) and in infective third-stage larvae (C, D). (A, B) strong GFP expression is found at the sphincter connecting pharynx and intestine (S). (C, D) GFP expressed in the pharynx muscle (PM) and body wall muscle (BM). Scale bars = 100 µm.

**Figure 7:**
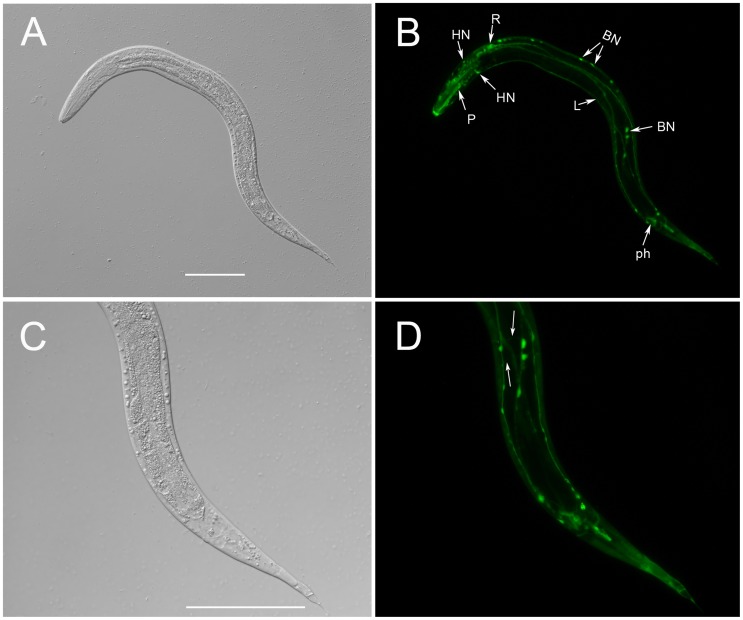
The spatial expression pattern of *Ss-riok-1* in the post-free-living first-stage and second-stage larvae. DIC (A, C) and fluorescence (B, D) images of transgenic *S. stercoralis* post-free-living L1-L2 stage larvae expressing *Ss-riok-1p::gfp::Ss-era-1t*. (A, B) GFP expression in the pharynx (P), head neurons (HN), body neurons (BN) and tail phasmidial neurons (ph) and longitudinal nerve tracts (L), GFP expression is also observed in the neurons (R) with positional homology to neurons of *C. elegans* in retrovesicular ganglion (RVG). (C, D) GFP expression in the commissures between body neurons and longitudinal nerve tracts (arrow). Scale bars = 100 µm.

To determine the anatomic expression pattern of *Ss-riok-1*, larval progeny of FL Female of *S. stercoralis* transformed with the construct pRP1 were screened for GFP expression. Some of the immature eggs had GFP expression, even when they were still in the vulva of the female adults (data not shown). After 24 h, newly hatched transgenic PFL L1s exhibited GFP expression throughout the body, with strongest expression at the boundary between pharynx and intestine (Fig. 6A and B). After 72 h, strong GFP expression under the *Ss-riok-1* promoter was seen in the nervous system, including some head neurons, body neurons and tail neurons as well as in pharynx and hypodermis of transgenic PFL L1s and PFL L2s (Fig. 7A and B). Processes of head neurons go through the body of these larvae, connecting to neurons in the body and tail. The nervous system of *S. stercoralis* has not been mapped in its entirety, so that a neural map of the free-living nematode *C. elegans* [50], [51] was employed as a model to tentatively identify of neurons expressing GFP under the *Ss-riok-1* promoter. Using this comparative approach, we concluded that body neurons expressing the *Ss-riok-1*-based reporter are likely sensory neurons and ventral nerve cord motor neurons (Fig. 7C, D, E and F). Furthermore, as PFL L1s developed towards iL3s in the next 4–5 days in culture at 22°C, *Ss-riok-1*-specific reporter expression was localized to zones in the body wall muscle of the parasite (Fig. 6 C and D).

## Discussion

The crucial role that RIOK-1 plays in the development of organisms was initially deduced from investigations in yeast as well as in *C. elegans* [8], [13]–[17]. In the present study, we laid the groundwork for functional studies of RIOK-1 in parasitic nematodes by isolating and characterizing the RIOK-1 encoding gene *Ss-riok-1* from *S. stercoralis*, an important parasite causing disease in humans and dogs.

The present study revealed only one *Ss-riok-1* transcript. By contrast, multiple *riok-1* transcript variants, with shortened C-terminal and N-terminal ends, have been identified in *C. elegans* and humans, respectively. The presence of only one *riok-1* transcript appears to be a common feature of parasitic nematodes as public database searches (results not shown) have failed to detect multiple *riok-1* transcript variants in various species including *A. suum*, *B. malayi*, *Dirofilaria immitis*, *H. contortus*, *L. loa*, *S. ratti*, *S. stercoralis*, *T. vitrinus* and *W. bancrofti*. The functional significance of transcript variants encoding an incomplete RIOK-1 in *C. elegans* and human is yet unknown.

The main functional domains in RIOK-1 appear to be conserved among organisms studied to date, including *Archaeoglobus fulgidus* and humans. Previous studies in yeast and human cells revealed that RIOK-1s have several functional domains possessing different functions. RIOK-1s lack the substrate binding motif commonly found in ePKs, but have a flexible loop (between β3 and αC) which is absent from ePKs [7], [52]. The conserved RIOK-1 signature sequence “STGKEA” in the ATP binding motif has higher similarity to the signature sequence “STGKES” in the ATP binding motif of RIOK-3 than to the analogous signature sequence “GxGKES” in RIOK-2. The Active site of RIOK-1 “LVHxDLSEYN” also has higher similarity to that of RIOK-3 “LVHxDLSExN” than to that of RIOK-2 “IHxDoNEFN”, and the two residues Asp (D) and Asn (N) in this motif are present in the active sites of all ePKs [7]. The active sites in ePKs are usually involved in the transfer of phosphate groups from adenosine triphosphate (ATP) to substrate proteins, and such phosphorylation events are basic to signal transduction pathways regulating numerous cellular and metabolic processes [5], [53]. Active site mutations that disrupt RIOK-1 kinase activity also interfere with recycling of two trans-activating factors (endonuclease hNobI and its binding partner hDim2) which are necessary for maturation of the human 40S ribosomal subunit [11]. Besides the active sites, the more divergent N-terminal and C-terminal regions of RIOK-1 also participate in some biological processes. The first 120 amino acids of the N-terminal region of human RIOK-1 interact with a complex consisting of protein arginine methyltransferase 5 (PRMT5) and methylosome protein 50 (MEP50), which are two components of the methylosome [54]. This RIOK-1-PRMT5 complex methylates the RNA binding protein nucleolin, which is involved in ribosomal maturation [55]–[58]. In addition to the active sites and the N-terminal region, the C-terminal region of yeast RIOK-1 is phosphorylated by the casein kinase 2 (CKII) to regulate the cell cycle in yeast [59]. The functions of the RIO domain and the N- and C-terminal regions of RIOK-1 in parasitic nematodes are unknown. In the present study, the predicted amino acid sequence alignment (Fig. 1) revealed that *Ss*-RIOK-1 shares common features with the RIOK-1 family. *Ss*-RIOK-1 has limited similarity in its N-terminal and C-terminal regions to yeast and human RIOK-1 homologues. In addition, *Ss*-RIOK-1 is capable of both phosphorylation and autophosphorylation, which is a property of the RIOK-1s from *A. fulgidus*, *S. cerevisiae* and humans [9], [11], [46]. Taken together, these findings suggest that *Ss*-RIOK-1 is an active protein kinase but its biological functions may differ from those of its homologues in yeast and humans.

*Ss-riok-1* contained fewer introns than its homologues from *C. elegans* and *H. contortus*. This reduction in intron number has been a consistent trend in comparisons of genes in *S. stercoralis* and their orthologs in *C. elegans* and its parasitic counterparts in clade V [21], [37], [60], [61]. The comparison of 5′-UTRs in *Ss-riok-1* and *Ce-riok-1* revealed some shared promoter elements, though the sequence similarity was limited. The promoter elements included TATA box, CAAT, GATA box and E-boxes were all found in the regulatory region of *Ss-riok-1* and *Ce-riok-1*. Along with the TATA box, the CAAT box is another common promoter element for protein-coding genes in eukaryotes [62]. The GATA box is recognized by GATA transcription factors and is necessary for regulation of eukaryotic development and reproduction [63]–[67]. E-boxes are recognized and bound by basic helix–loop–helix (bHLH) proteins which regulate a wide range of developmental process in eukaryotic organisms including neurogenesis and myogenesis [68]–[70]. 37 bHLH proteins have been identified in *C. elegans*, and some of them are associated with specification of neural lineages and differentiation of myogenic lineages [71], [72]. E-boxes are also characterized as gene promoter elements in the parasitic nematode *H. contortus* [73] and, as demonstrated here, in *S. stercoralis*, suggesting that these elements are involved in regulating the development of parasitic nematodes.

*Ss-riok-1* transcripts are present in all life stages of *S. stercoralis*, suggesting that this gene functions in the development of all stages of this parasite. The abundance of *Ss-riok-1* transcripts varies during development, being higher in the iL3 and in parasitic and post-parasitic life stages, which are progressing towards the free-living adults (FL Female and FL Male) than in the FL female and PFL L1. In order to explore the tissues that *Ss-riok-1* may function in *S. stercoralis*, the anatomical expression pattern is analyzed by employing transgenesis in free-living stages of this parasite.

The results of transgenesis showed that the GFP expression under the *Ss-riok-1* promoter occurs at embryos and PFL L1 suggesting that *Ss-riok-1* begins early in embryogenesis in the post-free-living life stages of *S. stercoralis*. *Ce-riok-1* expression in *C. elegans* occurs in a similar temporal pattern [74] and the embryos die when the expression of *Ce-riok-1* is silenced by RNAi, highlighting the essential role of *Ce-riok-1* in the embryogenesis of *C. elegans* [13], [17]. This embryonic lethality phenotype, along with the similar embryonic and early larval pattern of *Ss-riok-1* expression leaves open the possibility that this gene is also essential for embryogenesis in post-free-living stages of *S. stercoralis*.

As transgenic *S. stercoralis* developed from PFL L1s to PFL L2s in culture, strong GFP expression from the *Ss-riok-1*-based reporter persisted in the pharynx and nervous system (Fig. 7). With the exception of sensory neurons of the amphids and some related interneurons [75], [76], the nervous system of *S. stercoralis* has not been mapped in detail. Despite this, studies on the nervous system of *S. stercoralis* published do show how *C. elegans*, with its morphological similarities to free-living stages of *S. stercoralis* can be employed as a model to identify neurons in this parasite [76]. The *C. elegans* hermaphrodite contains 302 neurons including 282 neurons in somatic nervous system and 20 neurons in pharyngeal nervous system [51], [77]–[79]. There were 113 motor neurons that control crawling and swimming behaviours as well as the motility of the alimentary and reproductive systems [50], [51]. These motor neurons, along with some of the sensory neurons in the body, are connected by longitudinal nerve tracts and commissures [51]. The differentiation of *C. elegans* neurons begins at the proliferative phase of embryogenesis, and the nervous system mature at the late L1 and L2 stages. During the late L1 stage, some neurons, including five classes of ventral nerve cord (VNC) motor neurons, are generated from several cell lineages [78], [79]. GFP expression from our *Ss-riok-1*-based transcriptional reporter occurs in late L1–L2, in the dorsal cord (DC) and VNC which connect the neurons from head, body and tail. In addition to DC and VNC, the *Ss-riok-1* promoter is active in other longitudinal nerve tracts from head to tail and commissures sent by the body neurons and connected with DC in the PFL L1–L2 larvae of *S. stercoralis*. These findings suggest that *Ss-riok-1* contributes to the development and function of the nervous system of *S. stercoralis* during the development in PFL L1–L2.

Studies on the maturation of neurons in the mammalian central nervous system (CNS) [80]–[83] have shown that polyribosomes contribute prominently to the synaptogenesis, leading to protein synthesis. During the development of the neuronal system of *C. elegans*, neurons project axons, which reach their synaptic partners to establish complex neuronal circuits [84]. This process might rely on one or more molecular signals in a neuron to recognize the receptor molecule on synaptic partners [85]. In some organisms, polyribosomes accumulate in growing synapses and contribute to postsynaptic membrane specialization [86]. Considering the important role of RIOK-1 plays in the maturation of 40S ribosomal subunits in other eukaryotes [10], [11], *Ss*-RIOK-1, which our data suggest is localized in the nervous system of free-living stage larvae, could also participate in the development of this system in *S. stercoralis* through its function in ribosomal maturation and resulting support of protein synthesis in developing neurons and synapses.

In contrast to PFL L1s and PFL L2s, activity of the *Ss-riok-1* promoter is limited to the body wall of iL3. *Ss-riok-1* transcripts are also most abundant at this stage. Both of these findings are consistent with the fact that the cuticles of *S. stercoralis* iL3 undergo significant remodeling and that L3i become radially constricted overall in the transition from actively developing post-free-living stage larvae [3]. The presence of eight E-boxes in the 5′-UTR of *Ss-riok-1* is consistent with a role in myogenesis that may accompany morphogenesis of the highly motile *S. stercoralis* iL3 [71], [72]. Overall, the significant increase in abundance of *Ss-riok-1* transcripts in iL3 over PP L3 strongly suggested a pivotal role for *Ss*-RIOK-1 in the infective process and other aspects of parasitic life in iL3 of *S. stercoralis*. Localization of *Ss-riok-1* expression in body wall muscle, along with the findings from other systems implicating RIOK-1s in myogenesis suggest that the parasite RIOK-1 kinase may support the increase in motility that is essential for host-finding and contact by iL3.

The conservation in RIO domains between *Ss*-RIOK-1 and homologues from selected species suggests that *Ss*-RIOK-1 may participate in the ribosomal process in *S. stercoralis* as did its homologues in yeast and human cells [8]–[11]. Whether the kinase activity of *Ss*-RIOK-1 supports its function in the maturation of ribosome as well as the development of *S. stercoralis* is unknown. Transformation of *S. stercoralis* with a transgene construct encoding a kinase dead mutant *Ss*-RIOK-1 that interact with other proteins or ribosomal particles but can't function as an active kinase may disrupt the function of the endogenous *Ss*-RIOK-1, which could be an effective way to analyze the function of *Ss*-RIOK-1 in the development and growth of *S. stercoralis* [23], [24]. Understanding the function of RIOK-1 in regulating *S. stercoralis'* development may greatly help us to assess the potential of RIOK-1 as a drug target for the control of parasitic nematodes.

In conclusion, we have isolated and characterized the RIOK-1 encoding gene *Ss-riok-1* from the zoonotic parasite *S. stercoralis*. *Ss*-RIOK-1 contains a RIO1 signature motif and has high similarity to a range of homologues from different species. Recombinant *Ss*-RIOK-1 has kinase activity. *Ss-riok-1* transcripts are present throughout development in *S. stercoralis* with the highest abundance in iL3. The *Ss-riok-1* promoter is active in head neurons, body neurons and tail neurons as well as in pharynx and hypodermis of *S. stercoralis* of PFL L1s and PFL L2s and in body wall muscle of iL3. These findings suggest that *Ss-riok-1* plays an important role in regulating development of *S. stercoralis*, particularly in the formation of the nervous system in PFL L1 and L2s and in morphogenesis of the iL3 which is crucial to the infective process. Future work should focus on ascertaining whether *Ss*-RIOK-1 function is essential for the development or survival of *S. stercoralis* and by what mechanisms it exerts its function.

## Supporting Information

Figure S1 **Diagram of** ***Ss-riok-1*** **transcriptional reporter construct pRP1 used to transform** ***S. stercoralis*** **.** The 4280 bp promoter of *Ss-riok-1* was inserted into pAJ01 between the *Pst*I and *Age*I restriction sites. Length of *gfp* with artificial introns and *Ss-era-1* 3′ UTR are marked above them.
(DOC)

Figure S2 **Alignment of promoter regions predicted from the 5′-UTRs of** ***Ss-riok-1*** **and** ***Ce-riok-1*** **.** Coloured boxes represent the promoter elements: CAAT (CCAAT) or inverse CAAT (ATTGG) motif (turquoise), inverse GATA (TTATC) (green), inverse GATA (TTATC) (green); GC box (yellow); E-box (CANNTG) (grey); TATA box (pink). The number represents the position of the nucleotide upstream of the start codon.
(DOC)

Table S1 **The names and DNA sequences of primers used in the present study for isolating cDNA and promoter region of** ***Ss-riok-1*** **and for constructing protein expression and transgenic plasmids.**
(DOC)
