# Supplementary figures and images for: Toward Understanding the Functional Role of Ss-riok-1, a RIO Protein Kinase-Encoding Gene of Strongyloides stercoralis
Source: PLoS Negl Trop Dis. 2014 Aug 7;8(8):e3062. doi: 10.1371/journal.pntd.0003062 (PMC4125297; doi:10.1371/journal.pntd.0003062)

Fig S1


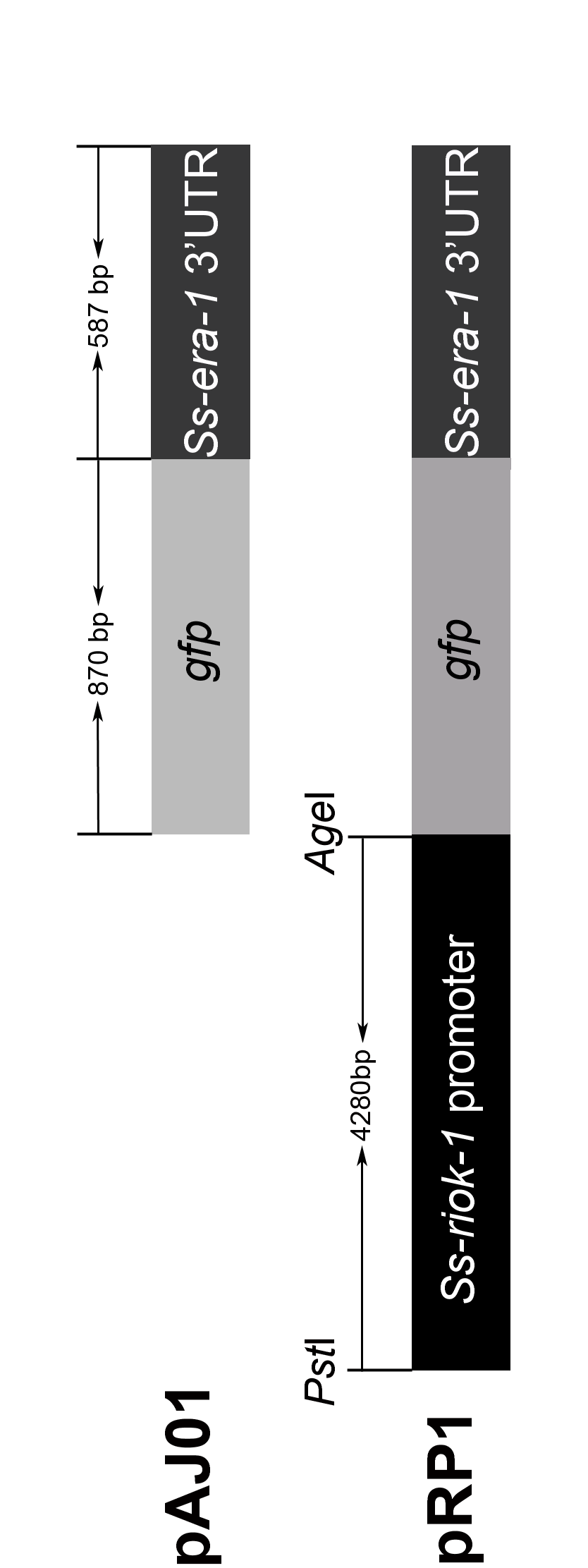

Supplement: Figure S1 — Diagram of Ss-riok-1 transcriptional reporter construct pRP1 used to transform S. stercoralis . The 4280 bp promoter of Ss-riok-1 was inserted into pAJ01 between the PstI and AgeI restriction sites. Length of gfp with artificial introns and Ss-era-1 3′ UTR are marked above them. (DOC) [file pntd.0003062.s001.doc]
