# Supplementary material for: Toward Understanding the Functional Role of Ss-riok-1, a RIO Protein Kinase-Encoding Gene of Strongyloides stercoralis
Source: PLoS Negl Trop Dis. 2014 Aug 7;8(8):e3062. doi: 10.1371/journal.pntd.0003062 (PMC4125297; doi:10.1371/journal.pntd.0003062)
Supplement: Figure S2 — Alignment of promoter regions predicted from the 5′-UTRs of Ss-riok-1 and Ce-riok-1 . Coloured boxes represent the promoter elements: CAAT (CCAAT) or inverse CAAT (ATTGG) motif (turquoise), inverse GATA (TTATC) (green), inverse GATA (TTATC) (green); GC box (yellow); E-box (CANNTG) (grey); TATA box (pink). The number represents the position of the nucleotide upstream of the start codon. (DOC) [file pntd.0003062.s002.doc]

Fig. S2

*Ss-riok-1* GTGTTATAAATTTAGGTCCCCTCTTTAAAGAGTGAATATTT---------AATGGAATATGGGGAGTAAAAATTTG--ATAAGACA-TATATCATTAGTATATAATATATATATTATACATATGTAATGTAACATTATAATGGACAATAAAGATTTCTTTAAAGCTAGAAAGAGA--AGT -4115

*Ce-riok-1* CGGCAAACCGGCAAATTGCC-------GAAAATGAAAATTTCTGGCAATCGGTAATTTGCCGAAAATTCAAATTTGTCGTAAACCAGCAAATCGCCGGAATTGAAAACTTGCGGCAAATCGGCAAAATGTCGCA-----------ATCAAAAATTTCCGCTAAACAGGCAAAAAATCGGC -4081

*. * . . *. * ** .*.*.**** **** ..*.. *.. *..*.* ******* .***. ** .* ***....* ** ** *. *... .* *. ... ***** .** * .***.*****. . **.* .* **.*.* .*.

*Ss-riok-1* TTACAATTT---ATTCTTAAGATATTTATTTATAATTATTGAGATA----TGTGTTCCTAAATGCCATTTATTTATCATTTATCTAACAAATAACTTTACTATCTTTTTTTTTCCCTAAATCATTATTATAATTTATCATTTTAACCCCTAGAATTTATCTATCACTCCACTATATTCCT -3942

*Ce-riok-1* CAAAAATATCAAAATCTTCAGATTTTTAAAAGTTATAAAAGAGAAAAAGGTATATTCCT--ATGCCAAAAAAAAGCATTTCTTACAATAGAAATCAGTTTTTCGCTCTTTTTTTGCAAAAT------------------TTTCAAACCCTAAAATTT--------------TGCCCATCC -3935

. * *** * * **** **** **** . ** * ***** *.*.***** ****** * .. **. * .**.*.* * * * .* . .*.******. * **** ***.** *****.***** *.. . .*.

*Ss-riok-1* TTAATATATATGATATGATATATAATGGAAAAAAAGACCAAAAAAAGTCAATAATGGTAAATAAATTGTATTCCATAACAAAAATAGATTTTTAATGTAATTATAGAAATGA-------------TTTATTACAAATACGAATAATATAACAAGATATGATTGAAATAATTTAAAAAATA -3775

*Ce-riok-1* CTGATGCAAATAGTG-------------------------------------------------------------AATTCAAATTGATTTTCGCT----TTGTAAATATGACACGTTCCTCCTCTTCCTCCTGTGTGCCATTGAT-CGATGAGACAGGAT------------------- -3840

.*.**..* **..*. **. **** ******.. * **.**.* **** **. *. .. .*.* * *.** ..*..***.* ***

*Ss-riok-1* TTTAGGATTTCCCTTTTATTATTAAAATTTCTTACT------------------ATTTATTCTTTATTTTTTAATTCTATTTTAAT--TTATTCCTATAATTTTGTCATTTCTTTACTAAAATTTATTTTACCTATTCAATTAATAAATTTAACATTATAGAAAGTACCAATAATATTAT -3615

*Ce-riok-1* ----GGCTCTCGTATTTAATACTTGATAGTCCAGTTTATGCACAGAGGGGGGGGGCTTCTCCTACATTTTTCAACAGCCGTTCGGTGCTCTCTCTCGGAGTTTTGTAA-------GCTGATACTGATACT--------AGTCGGAGGAGTTAAAATTTGAGGAATTTTCGTTTTCTTCAC -3679

** *.** . **** **.* .* **. ..* ..** *.** .******.**. . **...* *. .**... *.****** * .**.* *.* ** .* *.*... ..* **** *** **.** * .*. * . *.*.

*Ss-riok-1* AATACTATTTTTATAATACATACATAAGTTAGAAACAAAAGTTATCATGTGATAATCTAGTATAAATGTACATATATAAAAAAATCAAACGTCTAAATAATAAAGAATAAAAAATTAAAAAATTAAAATGTTTT----TTTTTAAAAAACTTTCTTACCATAATAATTTCACCACCACCA -3439

*Ce-riok-1* GAGAAAATTCGTAAAACCTGGAGTTTTCCTGGAAATTGAAATT----------------------------------------CTGAAATTTCCAAA-----------AAAAATGTGCAAAACCACAATTTGTCGAAATTTCCGGCAATTTCCGTTTTTCTGGCAAATTCGGCAATTC-- -3552

.* * ***. ** **. .. * * .*.****. .**.** * ***. **.*** ***** *. ****..* *** * *. ***.... ** .*.. ** .. *...** ***. ** . *

*Ss-riok-1* CTTGGAGAGGGGAGAAATGGGGGTGAAGTCGTGTTTATCAATAATAATATTTCACTAGAGAATGTGTCAAGAAAAATTATATATAATCAAACACTACTAC-------TGATATCAATGTTGTTTTTTCTATATTTAAAGCAACTGTGATAGACAGTATTCCAATTGTTGATATGGTTTTC -3266

*Ce-riok-1* ----------GCAGGTGTGCCGATTTGCCGGAATTTTTCAATTCCGGCAATTTGTC----GATTTGCCGGAAAAAATTGTCTGCCGTCCACCCCGAATACAAGTTCGTAAAACCAGGAGTTTTTTTTT--------------------------------------------CGATTTTT -3430

* **. .** *.* . . * .*** ***** ....* **.... .** **.*...*******.* *.. .** * * * * *** *.* *.**. . * ******. .*.****.

*Ss-riok-1* CTGACAAGATATTCTATAATTCTTTTTATTTTATTTATTTTTTTT------------TTGTAGGAAGATTATTTATTT----------------------------------CCTAGAAAAGGTTAGTATTTTTTTTAAAAAACTTTTT--TTTTATTGCTATAATAATTTATATTTTTT -3134

*Ce-riok-1* TCAAAGGAAAATTCTGGAAAC-------GTTCACTCGTCGTTTTTGAACACCGGAAATAGTGAAGAAATTATTCCATTGGAACGTGAGCCCGGCCTCGAAAAAAGTGAAAAGCCAAGAAATGGGCGGAGCTTATTTTCGGCAATTCTATAATTTGACAATTATCA-AGTTCAAACATTTT -3258

...* ...* *****. ** . **.*.*..*. ***** * **....*.******. ** ** ***** ** ..* ..** **** .. **.*.* * *** *. ..*** * *.**.* *. ****

*Ss-riok-1* -------------------------------------------------------TCTACATTTTTTAAAAATTT----TTTATTAAATTTAAAGTTAGTTTA------------------------------------------AAATTTAATAGTTATTAGTATGAATTAGAGATTAC -3055

*Ce-riok-1* GAAAAAAAAACTCGAAAAACTAAATTCCACAGAATTTTAGAAATAAATTCGGGAATCGGCATTCTCGGAAATTTTGCCGATTATTAAAATTTTCGATAATTCGGCATTTTTAGCAAAAACCAACGGCAAAATCGGCAAGTTCGACAAATCGGCTAATTGCCAGTTTG---CCGATTTTCC -3081

** .****.*. .*** *** ******** ** * **.**.. ****. . **.**...*** ** . ** ** *

*Ss-riok-1* GAAATTGACGTATGTTTACATTAATTTTTTTTATTCTAATGATAAAATATTTTT-------TTTACATAATAACTGTTTTGACTTATCTTTAAAAAGTGTAAAAAGATTTTTTTTGGTCATTTTGTTATTTAAAGTATTAGTTACTTTTATTTTT----AAATTTAATTAAATGC----- -2891

*Ce-riok-1* GAAAACAAAAAATATTTAAAAAAATTTTGCCGATTAAAATGATCTGAAATTTCCAAAAAAATGTGCATAACCACAAT------------------------------------------------TTGCCGAAAATATCGGCAATCGCCGTTTTTCCGGAAATTTGCCGATTTGCCAAAA -2949

**** ..* . **.**** * ****** .. *** ****** .* ****.. * *.*****. ** .* **... ***.***..*. *.. ...***** ******. . * ***

*Ss-riok-1* -AAATATTTTTATATGGTGCTTTTAAAATTAAAATCGA-----------TTTGAAAAA---------------------------GTCATTATTTGGACAAAAAAAAA------------GGTGTTTTTTTTTAATTT--ATAGTTTGCTTATTTTTTAATAATATTTAT---------- -2774

*Ce-riok-1* AAAATGTATTTTTAAAAAAATTTGACGATTAAAATTGAAGCTCTGAAATTTCCAAAAACAAAATTTGCCGAAAATTTCGGCGATTGCCTTTTTTCCGGTAAATCGAAGATATGCCGACTTGACGGAATTTTTCAATTCCGGCAATTTTCCGATTTGCCGAAAGAAATTATCTGCCGTCCA -2769

****.* *** ** .. . *** * .********.** **. ***** *.* ** **. *..*** .**. *..* *****.****. ..*.*** *. **** ...* *. * ****

*Ss-riok-1* ----------GAAAGTTATAATTTATTAAAGAAAATATTTTAAGAT-----------------AGTTTATCTATTATGTATAGTGATTATTT---------TAATAAATGCATCTATTTTTA--------------------GAAGTTTTATTAGTTTATAAATAAAAAAAAAATTATAA -2650

*Ce-riok-1* CCACAAATACAAAAGCTGTAAATTCTAAATAAAATCAATTCAATATTTCTTTAAATTTGTCGAAGTGTAGCTATCAGAAATAATTTCCATCTCCCACTCAATGACTAATAGCCTTCTTCTCATTTTCCCTCTTCCGCCGCCCCAAGTTTTCTGCCTTTGT-GATGAAAAAAGGACTGCGA -2590

.****.*.*** ** * ** .*** .* **.** ** *** ** ****.* . ***.* ..**.* *.*. ***. ..* **.*.* ******* * ***.* .**.******..*.*...*

*Ss-riok-1* ATTGTGGTAT------------------------------------------------AAATAGTTTAACATTTAAAGTAGCTAATTTTATATATTTATCATTTCATAAAATTGTAGCTTTAGGCTATGTTTTGCTCATTATAATGA--TTCATAGGAAAACAATTTTGATAGCCTATTA -2520

*Ce-riok-1* GTTTTAGTTTTCCAAGCCCGAATGAGTTTTCTTCCCCCCTTCAACGGTCGGGTCAATCGAGCAGAATAAC----GAAGAAGCTGCTCTGAAACTACTATTTTCTTGGGATTTTATTGGTTTTGAGTATCTTGAACTCTTTTTTTTGGTCTCCATTTCGCAATAATTTTGTTGTTTTTCTT -2414

.** *.** * .*..** **** .*** ****. *.* * *. .***. *.*.. .* **.* * *** *. *** ** .*** ** * **. *.*** . **.******* *. ..* .*

*Ss-riok-1* C---------------------------------AACTTTTTTCTACTATTA-----------ATAGTTTCATCCATTACTAAATAAACTTTTCATGTACTTTTTTTATATTCATATATTATTACATATCA-ATTTTTTCAAAGTATTTTTTT-------------------------AA -2410

*Ce-riok-1* CCGCATTATCTGAATCACCTGCAGAGGCAGTATTGATTTTTTTTAGTTTTTAGCGCAAAAAGTACAAGCTTAGAAGTTAGTCAATCAGTTTTTGTTGTA------------TAATTTATCA-TAGATATCACGTGGTGTCAAAGTGTCTCATTTCGGTTTGATCTACAAAGAATGCGGGA -2247

* .*.******. ..* *** *.*. .*.* .*** * *** *..**** **** * ** ***.* ** ****** .* * *******.*.*. ** .*

*Ss-riok-1* AAAAAAAAAAAGATGTTACAA-------CGTATGATGTAATTATTTTAAAAAGTATTTTTAAAAAAA-----ATATATATATATCAAGAAAAAGTAATATGTGTCATATGTATGTATATATTTAAACAGACATTCTTATTT---------TTTTAATATATTTTTTTATTATTGATATAT -2251

*Ce-riok-1* GAAGAGACGCAGACTTTTCAACTAATTTCGCATGGTTAAGAGCGCGTAACGGAAGTTTTCGCTGAAATTGGTATATTCAAGCATTCGAAGCTAAAGATGTCGGTTTTAAAAAAATATATAAACAACCGGAAATTATTACCCAGACGCGAAATTTAACACAGTACTCGGTC-----TCGAC -2072

.**.*.* . ***. ** *** **.***.* *. . *** ... .****.. .*** **** .* ..**. ..*. *. .**.* **. ** . * .****** .** *.** *** ***... *****.*.* * .*. .*. * *.

*Ss-riok-1* ATAAATATTCTAAGAGTTTAAATATGATCAATCATTTTGATTC-----TTTATTATTAAATTAAATAATAATATATTTTTTTATTTAAGA------TAGAAAGATTTGTACTACTTATACTTCCTCAAAATGAGTGTTTTCCTTTACCTGTCCCTATTATCATTGTTATTGAGAAGGTAA -2082

*Ce-riok-1* ACGACAATTTTGAGTACTGTAATTTCAAACCTCTTGTTGCTGCTGAATTTTGATATTTTTTTCGATGAAAATGTACTTTTTTAAATAAAAACTCAGATAAAATATTAAAAACACTTGAGAAATCGAATAAAAAA---------------------------------ATTCAGGAGGAAT -1925

*..* ***.*.** ..* *** * * ** * *** * * ***. **** ** .**.* ***.**.******* ***.* .*** *** . * .****. . .* * ** .*. *** **.*** *

*Ss-riok-1* AATAATTTAGAAAGTAGTAATATA----------TTTAACCTCTTATATCACAAATTGTTATAGTAAAAAAAAT-TATAATAATATC--------------------ATAATACGAATTTAATAGTATTGTTATGTAGACTTTTTTTTTTTC------TTATCATAAAGTAAGTATATGA -1939

*Ce-riok-1* AAGAGTTTGAAAATTCGTCGATTAGAGACCAGTTACCATACTTTTGGCTCAAAAATCGCCAAATTTACACGAATGAATAATAATTTCCGGTAATTTCTCTGATTTTTTAAATTAGAAAATATCAATTTTCTCATTTAAATCAACATTTTTTCGCAAATTTATTGGAAAAAAA----ATAA -1749

** *.***..*** * ** . ** ..* **.**. *** ****.*..* * * * * .*** ******** ** *** *** ** .*.* ** *.** **.*.. . ******* ****.. ***. ** **.*

*Ss-riok-1* AATGTGTTATAAATATATATTATAGT--AGAAATAGGTAAAAATTTCAAT------ATAATA---------AGTACTTTCTATACTAAGTATTTGAATATATAAAATGTTTTTGCAAAA---------------AAAATTCACTATATTATGAGGTATAGTACT---------------- -1794

*Ce-riok-1* AACAAAAAATAAGTAAATTTACTAATAAAAAAAAAGATACAAATTTTCATGATTGAATAATAATTTCCGGTAATATTTCTGATTTTTTAAATTAGAAAATATCAATTTTCTCATTGAAATAAACATTTTTTCGCAAAATTTATTGAAAAAAAAAGTCTAAAAATGAATTTTGTTAGCTTT -1576

**.. . ****.** ** * **.* *.*** **.** ******. ** ****** *.**.**.. ** .* . *** *** **** ** * *.*. ..*** ******.*.*. * * .*.** **. * *

*Ss-riok-1* ----------TTTTTTTATAGATTTCTTTATCCAACATTTTTATCACGTGTACCACACTATAGTATTACAATTACCAGA--AAAAAGAATTAGTTTAAGGACTCATATAC--AATTGATAAGTCCCCAGGTAAAAATGTTTTATT------TATTTATATTCTAAAT------------- -1660

*Ce-riok-1* AAAAAAAAAGTTTTTTTCAAAATTCTCCTATTACACATTTTTTT----TGATTTTCAGTATTTTGAAAATAGCTCCAAATTAAAAAAAAATTTTTTAACTATTTAAAAACGAAAATTTTAAATTTT---GTGAAAATATTTTTTTTTCCCCTTTCTGTACTCCCAGTGGACTTCTTCGTT -1396

******* *.***....***. ******** * ** .. ** *** *. * * . ***.* *****.** * ***** *.*.* * ** ** * ***.*... **.*****.**** ** * *.*.**.**. *.*

*Ss-riok-1* ---ATTCTCTATATGTCTAGTGTATGAGTCAAGCATAAAATAAAAAGTGGGATAAAAATACATATTCCTATGACTTTCATTATATTTTCTTACCTGGTTATAACTTTTTATGTATAGAGAAACATTATATGTATTTCCTTTTATAGTTTACTCTAGAATATTATTAAATATATTTTGTTT -1483

*Ce-riok-1* TGCACTTTCTCTGCCTCTGACTCATGTGTT--------------------------------CGCTGTTTGAATATTCAGTGCTTTTTCCCATTTTTCTTCTGCTTCTCCT--------------------CCCTCCCAAGTACACTTTACTACAAGTTTTCTCAGAGTTTTTCTCTTCT -1268

*.*.*** *.. ***... .*** **. ...* .* .*. **** *.. *****..*..* .* . .***.*. * . .*.** **.* ****** .*.. * *. . .*.* * *.*. *.*

*Ss-riok-1* TTATGTATTAAT--------TTATCTTTGACAGTTTTTAGTTTAATATATGGGATA-TATATTTAGAATTTATATAATATAAGTAAAGTGGATTATAAGGCT-------ATTGACATGCTTTCTATGTATATATATATATAATAAATATATATTTATATTATGGGTACTTAATATTATGA -1319

*Ce-riok-1* CCAAGTAACAGTAACAGGGCAAATATTTGACA--TCTTGTTCCAATTTGTCCGCCGCTACATTTGAA--CAAAACAACGCAATTAGTCGAGGTTTTACGGTTTTCCGAAATTGGACAGGTTTTAGGATATATATTCATAAAAT-GCCGTTTAACTCGTTTTTCGGTAATAAATATCCTG- -1094

..* *** .*.* ** ******* *.**. *..*** *.* * .. **.****..* . * *.**...** **. .*.** ** **.* ****. * ***. . .******* .*** *** . ..* ** .* ** * **** * *****. **

*Ss-riok-1* TGTGCCGTATATATTAAATAATTTTTGTGGTACCTGCTACATTATAAATAGATACTTTCTAGATAGTATATTTATTTTACAATGTTATTATTATTATTATTTCAAACCGATATATTCGATATCTCTATGGATTACCACATTGTCTCCGAAAAAAAAATAGATATATATATATATATTTAT -1139

*Ce-riok-1* ---------------------------------------------AAATATCTCTCCTCAAAATGGTGTGAAAA------------ATCGCAATAAAAGTTGCAAA---AATTGCCATATCTTTTTATTGGTTTTTATACAA---CCGAAAAAACAAAAAAAATG--------------- -992

***** * ...** *.**.**.*. * **... ** * .** **** * *... ** *.*.*** *.** ..*.*. . ********* ** *.* **.

*Ss-riok-1* GTTAATGTTAAGACAATGAATATTTT-----ATTTTTGAAGCTTTTTTAATGAAGAGCCT-----TTATATATCTATACAATAATGTTGTTTACTTGTTAAACTTTACCATTCTAGATTTTTGATGTCTATTTATTTAAAGGAAAAAAATGTTATAACTTTTTTTCCTACATGA------ -975

*Ce-riok-1* ------GTCTAGAAAATAGGGATTTTCATCCAATTTTAATGAATTTTTAACTAAAAATTTGAAAATTGTCTAGAAAAATTACATTTTCGGCTATTTTCCTCACTTAAACTAACAAAATATCTGAAAATTGTTT----------GGAAAATGCAA-------TTTGCCTATAAAAATTGGT -835

**. *** ***... ***** * ****.* * *******. **.*...* **.* ** * *. *.* * *.* .**.** .. **** * * * *.** *.*** . .*.*** ..******. * *** ****.* .*

*Ss-riok-1* --------ATATTTGTGAGAGTGATAAGGAAAGAAAAATTGAAAATGTTTAATCTTATGTATCATTTATTTAAATGTATTTAACAATAAGTTTTGTGATTCCTTTGAGATGGATATGTTGTATTGGTATCAATTTTAATTTTTCTTCTTTTTTTTTTATCTTCCAACTTCTTCTTTTAAA -803

*Ce-riok-1* TTTTAATTATTTTAATGAAAACGTTAAGGAAAAAGGCATTAAA-----------------ACAAATTTTTGAAATATTCAAAAACAGTATTTTCACCATTTCTCCGAA------------------------------------TTATTTTTTTGAAAAATTCAAACAT----------- -719

** ** .***.*..* ********.*.. ***.** *. * ** ** ****.* . ** * * ***... ***.**..**. ** ******* * *** *** *

*Ss-riok-1* TGGATAATAATAATAAGTTTTTGTAAGTTTTTTTTTTTCTGAATTATTATAGACATATATATATATATTTCTTTTAATA--------------------------TAATAAGATTTGTTTTTCATC---TCATAATACTTTTGTTAGAAATTTG------TTGAATTTATTCATTGAA-- -660

*Ce-riok-1* ----TAATCAAAAAACGTTTTCGGAAA---TTTTTTTTAGAAATAAACATTTCTAAAAATGCACATTTTTATCTTAAAAACTGATGAAAAATCGCTGAATAACGGCGAAAAAACATAATTTTCAGCTATTTCTCCTAGTTTATTCGAAAATTCGGTATTTTTAACATCAATTATCGAAAA -546

**** * ** * *****.* **. ******** .*** * .** .* * **..*.** *** *.**** * ..* **.*. *. ****** * *. * ** *** *...*****.* **.* *.* *.**.***

*Ss-riok-1* ----TTAGATAATATGAAATAGATATATTTTAACATGGCTAGTTT------TTGATATATTTTATT-----------------------GTATTGTAAAATGTTTTACATACAGAAAACTTTGAGTTTTGATGTACATATATATATATTATAGTTAACGAAAAATAAAGAAAAGATTTTT -513

*Ce-riok-1* TTAGGCAAAAAATTCCAAAAGTTTTCAGCTCAACATTGTCATTTTAATTGCTGAAAATTTTTTTTTTTAAAACCTCGAAACTTTGGTCAATAACTGGAAATGTTTTAAAT--AAAAATTTTGAAATTCTCGAATTTTTTTAAATATAAAAC-GTGAAAGTAACGCAGAAATTAGATTTTC -369

.*.* *** . *** . * .* .*.***** *..* *** * .* ** **** ** .** . .********** ** *.*** .** .*.**.* . .* . * ** ***** *. ** ** * ** ..*.*.* *******.

*Ss-riok-1* AG---------GAAGGAAGAATTTTTAAAAA--------ATTTTTAATGCCAATTGCTGCCACTGACTTAAGAGGAGGGGGGGGGGGGGAAGAAGAAAAGGATAGTTGAATTTTAGTCAAAAATACATCCATAAGCATATAGTTATTTATTGTTTTGTACTTAACTTTTATCCTTTTTAT -350

*Ce-riok-1* AGCTTAAAATCTCAAAAAAAAATTAGAAAAATCCAATTTCCCTTAAATTTCATTT-----------------------------------------AAAAAACTGAAAAATTTATTTCAAAAATTTTTCTGAAAATTTATTTCAAAAATTTTTCTGAAAATTTGGATTTCTCAAATTTTT -230

** *..**.** ** ***** ..** *** .** ** ***..*. * .***** ******** . **.. **.. *** . * ** *.* . * ** . *** ** *** *

*Ss-riok-1* TATATTTCTATGATATATAATATATGTGTAGGATCTATTATTATTATATATATTCAATATAAAGTACATGAAAAATTTTGATAACTTTAATATTATTTCTTTCTTTTCAACTTTTAGGGGGTGTTTTATACAATTATACTCTTAAATATAATATTTGTTTTATCAATTTTT-------AT -177

*Ce-riok-1* GTCTCCTCTACGTTCCATTATCTCTGCTCCTCACTCTCTCTCTCTCTCTCTCTC---------------------TCTCTCTCTCTCTCTCTCTCTCTCTCTCTCTTCTATATTCACGTGCCGT---------------------AGAGCGCACTTGTTTCGTAATCTTTCTCTCACGCT -92

. ..****.* * .** ** * **. . *... .* *. .* * * * *. *.*. * **.* . .* *.***.***.*** *. **.* * * .** * * ..*.******..* * .***. *

*Ss-riok-1* ATATAATTATATTATATTATTTTATATGTTATCTATTATTTCGGAAAAATTTAAAATTACAATATATTATTTATTTTATGATGGTGGAGGGGAAGATTATCAAGTATCAAAATTATAATTATTTATATTTTATAGGGTTATTTATATATATATACATCATATCTAAAAAGTGAAGG -1

*Ce-riok-1* GTCTCATCTTTTCATACTATTTCCTCGCTCTCATTTTTTCGCCCAAAAAT------------------------------------------------------AACCCAAATTTTTATTACGCGTGTTTCAGTGGGTAAC-------------------------------AAAC -1

.* * **. * *.***.*****. * *. . * ** *. * ****** *.* ***** * ****. ..*.***.* **** *. **.
