# Supplementary material for: Toward Understanding the Functional Role of Ss-riok-1, a RIO Protein Kinase-Encoding Gene of Strongyloides stercoralis
Source: PLoS Negl Trop Dis. 2014 Aug 7;8(8):e3062. doi: 10.1371/journal.pntd.0003062 (PMC4125297; doi:10.1371/journal.pntd.0003062)
Supplement: Table S1 — The names and DNA sequences of primers used in the present study for isolating cDNA and promoter region of Ss-riok-1 and for constructing protein expression and transgenic plasmids. (DOC) [file pntd.0003062.s003.doc]

| Primer names | Primer sequence |
| --- | --- |
| 1F | GGATGTATTTCAACAGGAAAGGAGGC |
| 2R | CCGCCCAAGTAGCGACCATCTTACG |
| 3F | CGGGATCGTTATGTGTCTGGAG |
| 4R | CCGCCCAAGTAGCGACCATCTTACG |
| 5F | AGTCTTAGATCCCCGTACCCGTC |
| 6R | ACAAGGCGAAAAATAATAAGACGGG |
| 7F | ACATAATCCTCGTAAGATGGTCGC |
| 8R | GGTCAAATTGTATAAAAGCTTCCCC |
| 10R | TTTCATGTGTAGGATAGTCGAGGTC |
| Ss-riok1-BamHI | CGGGATCCATGGTTGATATAGCAGAAAC |
| Ss-riok1-XhoI | CCTCGAGTTACGTTCTTTTTTTTGCATC |
| Ss-riok1-PstI | CGCTGCAGGTGTTATAAATTTAGGTCCCCTC |
| Ss-riok1-AgeI | CGACCGGTGAGAATGTTTCTGCTATATCAACCAT |
